# Reduction of Mean Arterial Pressure and Proteinuria by the Effect of ACEIs (Lisinopril) in Kurdish Hypertensive Patients in Hawler City

**DOI:** 10.5539/gjhs.v4n5p14

**Published:** 2012-06-30

**Authors:** Muslih A.I.

**Affiliations:** 1 College of Pharmacy, Hawler Medical University, Iraq

**Keywords:** kurdish race, ACEI, lisinopril, proteinuria, hypertension, ACEIs: angiotensin converting enzyme inhibitors, CRI: chronic renal insufficiency, ESRD: end-stage renal disease, RAS: renin-angiotesnsin system, ARB: angiotensin receptor blockers, MAP: mean arterial pressure

## Abstract

The angiotensin converting enzyme inhibitors (ACEIs) are a group of pharmaceuticals that are used primarily in treatment of hypertension and congestive heart failure, in some cases as the drugs of first choice. The renin-angiotensin system is activated in response to hypotension, decreased sodium concentration in the distal tubule, decreased blood volume and in renal sympathetic nerve stimulation. This study examines the effects of angiotensin converting enzyme inhibitor (Lisinopril) on blood pressure (BP) 131±2.4 and proteinuria 0.198±0.005 in Kurd hypertensive patients, mean arterial blood pressure and proteinuria excretion were measured weekly along the period of 12 weeks. Lisinopril significantly reduced mean arterial blood pressure, and attenuated proteinuria level in patients subjected to this study in lisinopril 10mg dose dependent manner (p<0.05, n=24). In conclusion, lisinopril is of beneficial of renoprotection and in lowering BP

## 1. Introduction

Elevated blood pressure and severe proteinuria are important predictions of progressive renal injury (Yano et al., 2012). How proteinuria results in tubulointerstitial injury, single strongest determinant of the long-term loss of glomerular filtration rate leading to end-stage renal disease, is incompletely understood (Theilig, 2012; Izu et al., 2012).

The excretion of protein in the urine (proteinuria) is generally thought to be an indicator of deteriorating kidney function. While drugs which lower high blood pressure (hypertension) may all contribute to the preservation of kidney function, experiments in diabetic rats with hypertension have shown that the drugs which function by inhibiting angiotensin-converting enzyme are more effective in reducing proteinuria than other antihypertensive drugs (Windt et al., 2008).

Urinary excretion of albumin is a sign of mild kidney disease (Brantsma et al., 2008). There is clear evidence that pharmacologic blockade of the renin-angiotesnsin system (RAS) with angiotensin-converting enzyme inhibitors (ACEIs) or angiotensin receptor blockers (ARB) reduces proteinuria and slows the progression of renal disease in diabetic and nondiabetic nephropathies, a beneficial effect that is not related to blood pressure control. Some patients exhibit a significant beneficial response, whereas others do not. The absence of response may be explained by the incomplete blockade of the RAS obtained with ACEI (Fernand-Juarez et al., 2006).

Intervention in the renin-angiotensin system (RAS) with angiotensin-converting enzyme inhibitors (ACEIs) is the therapy of choice for proteinuric renal disease, since these drugs lowered blood pressure (BP) and proteinuria and preserve renal function in the long term (Taal, 2000; Jafar et al., 2001; Van der Wonder et al., 2005). It is generally thought that reduction in the formation of angiotensin II (Ang II) is the main pharmacological action of ACEI. However, evidence is growing that other components of the RAS may contribute to the beneficial effects of ACEI (Carey & Siragy, 2003) in particular, angiotensin 1-7 (Ang 1-7), circulating levels of which are increased 10- to 25- fold during ACEI therapy (Liu et al., 2010; Li et al., 2011). These increased Ang (1-7) levels are thought to contribute to the antihypertensive effect of ACEI (Ferrario et al., 1997). The present study is designed to investigate the antihypertensive and renoprotective effects of lisinopril after a period of 12 weeks treatment in Kurd hypertensive patients.

## 2. Materials and Methods

This research was performed on randomly chosen hypertensive patients (all patients with other chronic diseases were excluded), the treatment period lasted three months, during which 24 patients; 11 males and 23 females were taking lisinopril 10mg/day. Baseline measurements of BP, is taken, and at 1, 3, 5, 7, 9, and 11 weeks of treatment. Urine samples were collected from patients on day 1 prior to first dosing with antihypertensive therapy (baseline) and at 2, 4, 6, 8, 10, 12 weeks of the treatment period of time. Serum creatinine level was measured before treatment and after one week of it, the result was within the normal range: patients on (ACEÍs) or (ARBs), their serum creatinine and after one week of starting treatment should not be exceeding more than 30% of their baseline value, otherwise it may precipitate unilateral and/or bilateral artery stenosis which lead to its exclusion (Fisher & Williams, 2005).

### 2.1 Parameters Measured

BP was determined according to Riva Rocci (Verrij et al., 2008; Eeftinck et al., 2009), by two measurements in the sitting position after 5 minutes at rest. All the measurements were made by the same investigators on the patient’s dominant arm between 8 a.m. and 11 a.m. MAP was calculated as (Razminia et al., 2004):


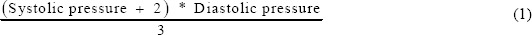


### 2.2 Laboratory Methods

24-hr urine samples were collected by spontaneous voiding urineprotein excretion was determined by spectrophotometer (CECIL CF 2021, England), Autoanalyzer (Hitachi, Mito, Japan)

### 2.3 Statistical Analysis

Statistical Analysis was performed using the ANOVA with respect to mean arterial pressure (MAP), and proteinuria. Differences between two measurements within one group were tested by *t*-test for dependent samples.

## 3. Results

First part of the results is revealing the effect of lisinopril on MAP in patients with essential hypertension. It is evident from the Table 1 that lisinopril causes a significant reduction in mean arterial pressure at 1, 3, 5, 7, 9, and 11 weeks after treatment compared to zero time values. After 11 weeks of treatment, the mean arterial pressure was 97±0.9 mmHg (25.9% decreased).

**Table 1:** Effects of lisinopril (10 mg) on Mean Arterial blood pressure in patients with essential hypertension at different time interval

Duration (week)	Mean Arterial Pressure (MAP)(mmHg)
0	131±2.4
1	122*±2.1
3	115*±1.6
5	109*±1.3
7	104*±1.1
9	99*±1
11	97*±0.9

* Significant at p<0.05 compared with zero time value

Values are presented as mean ± standard error

Patient number is 24

**Figure 1:**
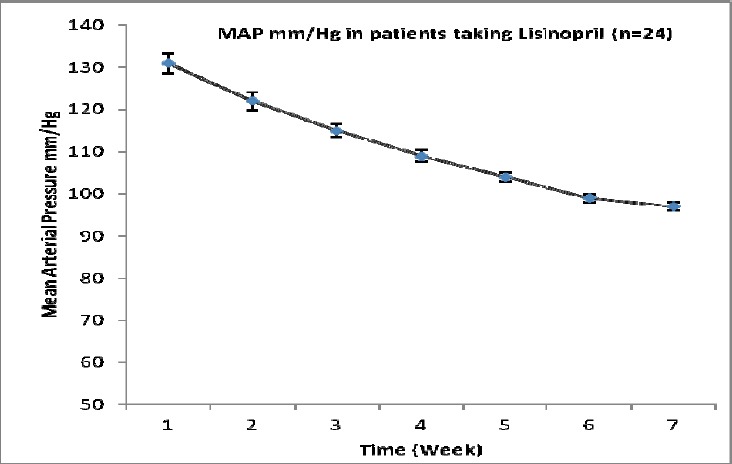
Effects of lisinopril (10 mg) on mean arterial blood pressure in patients with essential hypertension at different time interval; at baseline and after 3 months follow-up

The second part is the effect of lisinopril on proteinuria in patients with essential hypertension. Table 2 showed the effect of lisinopril on proteinuria in patients with essential hypertension at different time after treatment. There is significant reduction in proteinuria at 10 and 12 weeks (3 months) after treatment compared to zero time values. After 12 weeks (3 months) of treatment, the proteinuria decreased from 0.1985±0.00518 G/24hr to 0.1463*±0.00310 G/24hr (p<0.05) (26.2% decreased).

**Table 2:** Effects of lisinopril (10 mg) on proteinuria in patients with essential hypertension at different time interval

Duration (week)	Proteinuria (G/24 hr)
0	0.1985±0.00518
2	0.1898±0.00489
4	0.1784±0.00412
6	0.1712±0.00395
8	0.1643*±0.00332
10	0.1529*±0.00308
12	0.1463*±0.00310

* Significant at p<0.05 compared with zero time value

Values are presented as mean ± standard error

Patient number is 24

**Figure 2:**
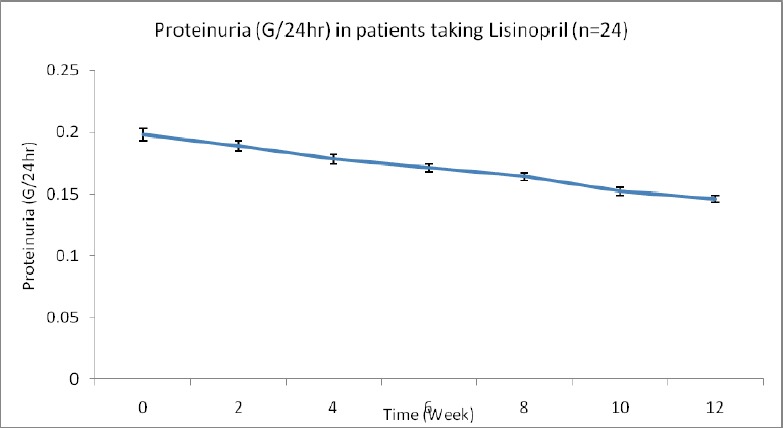
Effects of lisinopril (10mg) on proteinuria in patients with essential hypertension at different time interval; at baseline, and after 3 months’ follow-up

## 4. Discussion

Residual proteinuria is a strong modifiable risk factor for renal failure progression (Esnault et al., 2010). Hypertension and diabetes are the two biggest factors for proteinuria. Age and weight gain also increases the risk.

The remaining risk of progression of hypertension kidney disease to end-stage renal disease (ESRD) is still high despite introduction of diverse categories of new drugs (Koya et al., 2011). The present study shows the importance of targeting remission and regression of proteinuria in hypertensive patients. In our experimental study positive remission was obtained (proteinuria decreased from 01985±0.005G/day at onset to 0.1463±0.003 G/day at follow up.

The role of blockade of the rennin-angiotensin system has been explored in many clinical contexts with positive results (Lewis et al., 2001; Mendizábal-Ruiz et al., 2011). A number of observational and experimental studies revealed that angiotensin converting inhibitors (ACEI) delay the progression of renal abnormalities in patients with chronic renal insufficiency (CRI) and this protective ability is found to be mediated mainly by antihypertensive (systemic) and antiproteinuric (introglomerular) mechanisms (Rossing et al., 2003). Researches which conducted in various centers showed that dual blockade of the rennin-angiotensin system had little reducing effect on reducing proteinuria or renal function in diabetic hypertensive patients (Jafar et al., 2001; Cinotti & Zucchelli, 2001; Remuzzi et al., 2002; Brenner et al., 2003; Kunz et al., 2008; Messerlil et al., 2010).

By contrast, positive effects were observed in a trial of four weeks duration in normotensive subjects with nephropathy (Mogensen et al., 2000).

We evaluated the antihypertensive and renoprotective effects as reflected by short-term changes in blood pressure and in proteinuria. Across past two decades ACEIs expressed powerful effects in lowering blood pressure by inhibiting production of angiotensin II and impeding breakdown of bradykinin to which the cough is attributed after administration of lisinopril (Li et al., 2008).

The results in this study show that inhibition of the rennin-angiotensin system is particularly effective in lowering mean arterial pressure to about normal values 97±0.9 in Kurdish patients after one week of the treatment. This work provides evidence of an important role for lisinopril therapy in Kurd patients with hypertension as this remedy is effective at reducing mean arterial blood pressure and has beneficial effect on proteinuria in Kurd patients after 8, 10, and 12 weeks of medication, a result which is parallel to that documented by many other researchers (Chaturvedi et al., 1998; Zhuo et al., 2011). This study supports a potentially high beneficial medication approach for the treatment of hypertension and a protective tool from diabetic renal disease (Wienen et al., 2001).

## References

[ref1] Brantsma A. H, Bakker S. J. L, Hillege H. L, de Zeeuw D, de Jong P. E, Gansevoort R. T (2008). for the PREVEND Study Group. Cardiovascular and renal outcome in subjects with K/DOQI stage 1–3 chronic kidney disease: the importance of urinary albumin excretion. Nephrol. Dial. Transplant.

[ref2] Brenner B. M, Zagrobelny J. A (2003). Clinical renoprotective trials involving angiotensin II-receptor antagonists and angiotensinconverting-enzyme inhibitors. Kidney Int.

[ref3] Carey R. M, Siragy H. M (2003). Newly recognized components of the rennin-Angiotensin system: potential roles in cardiovascular and renal regulation. Endocr. Rev.

[ref4] Chaturvedi N, Sjolie A-K, Stephenson J. M, Abrahamian H, Kelpes M, Castellarin A (1998). The EUCLID Study Group. Effect of lisinopril on progression of retinopathy in people with type 1 diabetes. Lancet.

[ref5] Cinotti G. A, Zucchelli P. C (2001). Collaborative Study Group. Effect of Lisinopril on the progression of renal insufficiency in mild proteinuric non-diabetic nephropathies. Nephrol. Dial. Transplant.

[ref6] Eeftinck Schattenkerk D. W, van Lieshout J. J, van den Meiracker A. H, Wesseling K. R, Blanc S, Wieling W, Westerhof B. E (2009). Nexfin noninvasive continuous blood pressure validated against Riva-Rocci/Korotkoff. Am. J. Hypertens.

[ref7] Esnault V. L. M, Ekhlas A, Nguyen J. M, Moranne O (2010). Diuretic uptitration with half dose combined ACEI + ARB better decreases proteinuria than combined ACEI + ARB uptitration. Nephrol. Dial. Transplant.

[ref8] Fernandez-Juárez G, Barrio V, de Vinuesa S. G, Goicoechea M, Prage M, Luňo J (2006). Dual Blockade of the Renin-Angiotensin System in the progression of Renal Disease: The Need for More Clinical Trials. J. Am. Soc. Nephrol.

[ref9] Ferrario C. M, Chappell M. C, Tallant E. A, Brosnihan K. B, Diz D. I (1997). Counter regulatory actions of angiotensin (1-7). Hypertension.

[ref10] Fisher N. D. L, Williams G. H, Kasper DL, Brawnwald E, Fauci AS, Hauser SL, Longo DL, Jameson JL (2005). Hypertensive vascular disease IN: Harrisson’s principles of internal medicine.

[ref11] Izu A, Sugimoto K, Fujita S, Nishi H, Takemura Y, Okada M, Takemura T (2012). Nonfunction of the ECT2 gene may cause renal tubulointerstitial injury leading to focal segmental glomerulosclerosis. Clin. Exp. Nephrol.

[ref12] Jafar T. H, Schmid C. H, Landa M (2001). Angiotensin-converting enzyme inhibitors and progression of nondiabetic renal disease. A meta-analysis of patient-level data. Ann. Intern. Med.

[ref13] Jafar T. H, Stark P. C, Schmid C. H, Landa M, Maschio G, Marcantoni C (2001). for the AIPRD Study Group. Proteinuria as a modifiable risk factor for the progression of non-diabetic renal disease. Kidney Int.

[ref14] Koya D, Araki S-i, Haneda M (2011). Therapeutic management of diabetic kidney disease. J. Diabetes Invest.

[ref15] Kunz R, Friedrich C, Wolbers M, Mann J. F. E (2008). Meta-analysis: Effect of Monotherapy and Combination Therapy with Inhibitors of the Renin–Angiotensin System on Proteinuria in Renal Disease. Ann. Intern. Med.

[ref16] Lewis E. J, Hunsicker L. G, Clarke W. R, Berl T, Pohl M. A, Lewis J. B, Raz I (2001). Renoprotective effect of the angiotensin-receptor antagonist irbesartan in patients with nephropathy due to type 2 diabetes. N. Engl. J. Med.

[ref17] Li E, Li S, Yang W (2011). Enalapril, irbesartan, And Angiotensin- (1-7) Prevent Atrial Tachycardia-Induced Electrical Remodelling. Difficult Decisions In Precutaneous Myocardial Revascularisation – LIVE FROM FRANCE.

[ref18] Liu E, Yang S, Xu Z, Li J, Yang W, Li G (2010). Cardiac Resynchronization Therapy and Beyond. Angiotensin-(1-7) prevents atrial fibrosis and atrial fibrillation in long-term atrial tachycardia dogs.

[ref19] Li P, Kondo T, Numaguchi Y, Kobayashi K, Aoki M, Inoue N, Murohara T (2008). Role of Bradykinin, Nitric Oxide, and Angiotensin II Type 2 Receptor in Imidapril-Induced Angiogenesis. Hypertension.

[ref20] Mendizábal-Ruiz A. P, Morales J, Martinez X. C, Gutierrez Rubio S. A, Valdez L, Vásquez-Camacho J. G, … Moran Moguel M. C (2011). RAS polymorphisms in cancerous and benign breast tissue. Journal of Renin-Angiotensin-Aldosterone System.

[ref21] Messerli F. H, Staessen J. A, Zannad F (2010). Of fads, fashion, surrogate endpoints and dual RAS blockade. Eur. Heart J.

[ref22] Mogensen C. E, Neldam S, Tikkanen I, Oren S, Viskoper R, Watts R. W (2000). … for the CALM study group. Randomised controlled trial of dual blockade of renin-angiotensin system in patients with hypertension, microalbuminuria, and non-insulin dependent diabetes: the candesartan and lisinopril microalbuminuria (CALM) study. BMJ.

[ref23] Razminia M, Trivedi A, Molnar J, Elbzour M, Guerrero M, Salem Y, Lubell D. L (2004). Validation of a new formula for mean rterial pressure calculation: The new formula is superior to the standard formula. Catheterization and Cardiovascular Interventions.

[ref24] Remuzzi G, Ruggenenti P, Perico N (2002). Chronic Renal Diseases: renoprotective benefits of renin-angiotensin system inhibition. Annals Int. Med.

[ref25] Rossing K, Jacobsen P, Pietraszek L, Hans-Henrik P (2003). Enoprotective effects of adding angiotensin ii receptor blocker to maximal recommended doses of ace inhibitor in diabetic nephropathy. Diabetes Care.

[ref26] Taal M. W, Brenner B. M (2000). Renoprotective benefits of RAS inhibition: From ACEI to angiotensin II antagonists. Kidney Int.

[ref27] Theilig F (2012). Spread of glomerular to tubulointerstitial disease with a focus on proteinuria. Ann Anat.

[ref28] Van der Wonder E. A, Henning R. H, Deelman L. E, Rocks A. J. M, Boomsma F, de Zeeuw D (2005). Does Angiotensin (1-7) Contribute to the Antiproteinuric Effect of ACE inhibitors?. J. Renin-Angiotensin Aldosterone Syst.

[ref29] Verrij E, van Montfrans G, Bos J. W (2008). Reintroduction of Riva-Rocci measurements to determine systolic blood pressure?. Neth J Med.

[ref30] Wienen W, Richard S, Champeroux P, Chantal A-G (2001). Comparative antihypertensive and renoprotective effects of telmisartan and lisinopril after long-term treatment in hypertensive diabetic rats. JRAAS.

[ref31] Willemijn A. K. M, Windt Henning R. H, Kluppel A. C. A, Xu Y, de Zeeuw D, van Dokkum R. P. E (2008). Mayoca-Dial infarction does not further impair renal damage in 5/6 nephro-Ctomized rats. Nephrol Dial Transplant.

[ref32] Yano Y, Sato Y, Fujimoto S, Konta T, Iseki K, Moriyama T, Watanabe T (2012). Association of High Pulse Pressure With Proteinuria in Subjects With Diabetes, Prediabetes, or Normal Glucose Tolerance in a Large Japanese General Population SampleAssociation of High Pulse Pressure With Proteinuria in Subjects With Diabetes, Prediabetes, or Normal Glucose Tolerance in a Large Japanese General Population Sample. Diabetes Care.

[ref33] Zhuo M, Dong J, Zheng Y, Zuo L (2011). Are ACEI/ARBs associated with the decreased peritoneal protein clearance in long-term PD patients?. Nephrol. Dial. Transplant.

